# Oncogenic *PIK3CA* Mutation and Dysregulation in Human Salivary Duct Carcinoma

**DOI:** 10.1155/2014/810487

**Published:** 2014-01-08

**Authors:** Wanglong Qiu, Guo-Xia Tong, Andrew T. Turk, Lanny G. Close, Salvatore M. Caruana, Gloria H. Su

**Affiliations:** ^1^The Department of Pathology, Columbia University Medical Center, 1130 Saint Nicholas Avenue, ICRC 10-04, New York, NY 10032, USA; ^2^Herbert Irving Comprehensive Cancer Center, Columbia University Medical Center, 1130 Saint Nicholas Avenue, ICRC 10-04, New York, NY 10032, USA; ^3^The Department of Otolaryngology and Head and Neck Surgery, Columbia University Medical Center, 1130 Saint Nicholas Avenue, ICRC 10-04, New York, NY 10032, USA

## Abstract

Salivary duct carcinoma (SDC) is an aggressive malignant tumor with a high mortality, which resembles high-grade breast ductal carcinoma in morphology. The parotid gland is the most common location. Its molecular genetic characteristics remain largely unknown. We have previously reported high incidence of *PIK3CA* somatic mutations in head and neck squamous cell carcinoma, particularly in pharyngeal cancers. Here we examined the *PIK3CA* gene expression status and hotspot mutations in six cases of SDC by immunohistochemistry and genomic DNA sequencing. Immunohistochemistry showed that *PIK3CA* expression was elevated in all six patients with SDC. By DNA sequencing, two hotspot mutations of the *PIK3CA* gene, E545K (exon 9) and H1047R (exon 20), were identified in two of the six cases. Our results support that oncogenic *PIK3CA* is upregulated and frequently mutated in human SDC, adding evidence that *PIK3CA* oncogenic pathway is critical in the tumorigenesis of SDC, and may be a plausible drug target for this rare disease.

## 1. Introduction

Salivary duct carcinoma (SDC) was named based on its close resemblance to breast ductal carcinoma in morphology [1]. Most of SDCs are located at the parotid gland, and males are diagnosed approximately three times more frequently than females with SDC [2]. SDC is a rare tumor which only accounts for less than 5% of all head and neck cancer and 1–3% of all salivary gland tumors [3, 4]. The survival of patients with SDC is poor, with most dying within three years [5, 6]. Conventional treatments for SDC patients such as surgery with or without radiotherapy usually lead to high recurrence rate [7]. Therefore, novel treatment methods including targeting chemotherapy in combination with postoperative radiotherapy would be desirable [8]. However, little is known about the molecular profile of this rare disease.

The phosphatidylinositol 3-kinase (PI3K) signaling pathway is involved in many critical cellular processes, such as cell proliferation and survival [9]. Genetic alterations in the key components of the PI3 K pathway have been identified in diverse human tumors, including the *PIK3CA* gene [10, 11]. *PIK3CA*, which is the catalytic subunit p110 *α* of PI3-kinase, has been demonstrated to play an oncogenic role in some human cancers *in vivo* and* in vitro *[12]. Somatic mutations of the *PIK3CA* gene have been also reported frequently in numerous cancer types including head and neck cancers [13–17]. Most of these reported mutations are clustering in the exons 9 and 20 of the *PIK3CA* gene, where three hotspot mutations (E542 K, E545 K, and H1047R) reside. All those three *PIK3CA *hotspot mutations have been proven to be oncogenic and are associated with poor clinical outcomes [18, 19].

We have previously reported high incidence of *PIK3CA* somatic mutations in head and neck squamous cell carcinoma [13, 14], particularly in pharyngeal cancers [15]. However, the *PIK3CA* mutation status in patients with SDC was not included in that study. Furthermore, SDC shares many similarities with breast ductal carcinoma both in histology and biology, in which frequent *PIK3CA* mutation has been identified in human breast cancer [20]. Based on these observations, we investigated the PIK3CA protein expression and genetic mutation in six SDC patients by immunohistochemistry (IHC) and direct genomic DNA sequencing. The results showed that PIK3CA expression was elevated in all six salivary ductal carcinomas; 2 of them were identified with *PIK3CA* hotspot mutations.

## 2. Materials and Methods

### 2.1. Patient Samples

Acquisition of tissue specimens was approved by the Columbia University Medical Center (CUMC) Institutional Review Board and performed in accordance with Health Insurance Portability and Accountability Act (HIPAA) regulations. A total of six cases of SDC were identified from the archival tissues banked between 1997 and 2012 at the CUMC. The cases were reviewed by two pathologists with expertise in head and neck pathology (Guo-Xia Tong and Andrew T. Turk), and the diagnoses were confirmed.

### 2.2. Immunohistochemistry

Unstained 5 micron sections were cut from the paraffin blocks of SDC cases and deparaffinized by routine techniques. Tissue sections were treated with 0.01 M tri-sodium citrate buffer and boiled in a microwave for 15 minutes. Slides were then cooled for 10 minutes in tap water before blocking with Dako peroxidase blocking reagent (Catalogue no. S2001, Dako, CA). Primary antibody anti-PI3 kinase p110 *α* (Cell Signaling, MA) was diluted at 1 : 10 and incubated at room temperature for one hour. For HER2 staining (Epitomics), the condition was 1 : 250 dilution of the primary antibody and incubation at room temperature overnight. Then, Dako LSAB + System-HRP kit (Catalogue no. K0690, Dako, CA) was used by adding biotinylated link universal and streptavidin-HRP, each for 15 minutes at room temperature. Sections were counterstained with hematoxylin. IHC results were scored by the two pathologists with scales from 0 to 2+.

### 2.3. DNA Extraction from Tissue Specimens

Tumor and adjacent normal tissue were separately microdissected from 10 micron sections cut from each patient's paraffin block. Genomic DNAs were extracted using DNeasy tissue kit (QIAGEN Inc., CA). The procedures were performed according to the manufacturer's instructions.

### 2.4. Genomic DNA Sequencing

Exons 9 and 20 of the *PIK3CA* gene were analyzed by PCR amplification of genomic DNA and direct sequencing of the PCR products [14]. Specific primers for the *PIK3CA* gene exons 9 and 20 (PIK-E9F: CCAGAGGGGAAAAATATGACA; PIK-E9R: CATTTTAGCACTTACCTGTGAC; PIK-E20F: CATTTGCTCCAAACTGACCA; PIK-E20R: GGTCTTTGCCTGCTGAGAGT) were designed for efficient PCR amplification from paraffin-embedded specimens. PCR products were purified using the Geneclean Turbo Nucleic Acid Purification Kit (QIAGEN, CA). Finally, purified DNA fragments were directly sequenced using the corresponding forward PCR primers. Samples found to have a genetic alteration in the target exon were subsequently sequenced in the reverse direction to confirm the mutation using the reverse PCR primers. The mutation was then further verified by sequencing of a second independently amplified PCR product from the original genomic DNA template. All sequencings were performed with ABI's 3100 capillary automated sequencers at the DNA facility of Columbia University Medical Center in New York [14].

### 2.5. Quantitative PCR for Measurement of PIK3CA Amplification

 The genomic copy numbers of the *PIK3CA* allele were analyzed by quantitative real-time PCR measurement of each tumor and corresponding normal tissues. Specific primers (PIK-qF: TGCAAAGAATCAGAACAATGCC; PIK-qR: CACGGAGGCATTCTAAAGTCA) were designed for this genomic real-time PCR of *PIK3CA* encoding allele from paraffin-embedded specimens. Beta-actin was chosen as the reference gene for this assay (ACTB-qF: TAGAAGCCTCTTCATGGACAAC; ACT-qR: GTATCAGGCATGCAACACAAG). The SYBRGreen reagents applied for the real-time PCR were purchased from life technologies. 

## 3. Results

### 3.1. Clinical and Pathologic Findings

The SDC patients in this study show a median age of 62 years (range, 50–71) and a high male to female ratio (5 : 1). All the SDC cases occurred in the common parotid gland and the tumor sizes ranged from 1.6 cm to 2.3 cm. Histologically, the tumors showed both intraductal and invasive components. The intraductal component had a predominantly cribriform architecture and prominent comedo-type necrosis. The invasive components consisted of irregular infiltrative nests of tumor cells surrounded by prominent fibrosis and sclerosis. The tumor cells had abundant eosinophilic, somewhat granular cytoplasm, and enlarged, mildly pleomorphic nuclei with single prominent nucleoli. Mitotic activity and necrosis were variably present. Perineural invasion was present in all invasive cases (100%) and vascular invasion in four (67%). Evidence of a preexisting pleomorphic adenoma (carcinoma ex-pleomorphic adenoma) was seen in 3 cases (50%), one (SDC5) of which consisted of *in situ* salivary duct carcinoma where the malignant cells were confined within the capsule of the preexisting pleomorphic adenoma. Metastases to lymph nodes were present in 4 out of 6 cases (67%), where lymph nodes were removed. The clinical and pathologic findings are summarized in Table 1.

### 3.2. PIK3CA Expression Was Elevated in Salivary Duct Carcinoma

**Figure 1:**
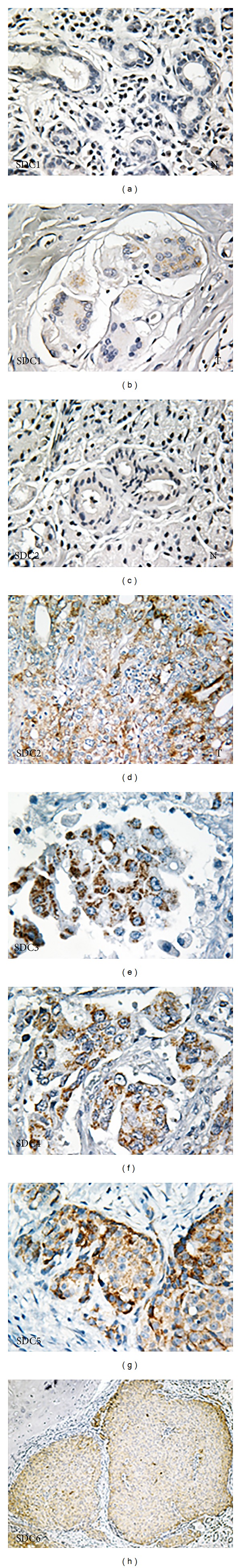
Immunohistochemical analysis of salivary duct carcinoma with anti-PIK3CA antibody. Representative PIK3CA IHC results of adjacent normal (a) and (c) and tumor tissues ((b) and (d) to (h)) from SDC patients. Staining of neoplastic cells was of greater overall intensity ((b) and (d) to (h)) relative to staining of nonneoplastic cells (a) and (c). Magnification: 200x.

Six specimens of SDC lesions were immunolabeled with anti-PI3 Kinase p110 *α* (PIK3CA) antibody to assess the expression level of PIK3CA proteins. IHC results showed focal cytoplasmic positivity in tumor cells compared to normal ductal epithelial cells. Although elevated PIK3CA expression was also observed in some normal ductal epithelial cells of salivary glands immediately surrounding the tumor masses, immunolabeling of the neoplastic cells was of much greater overall intensity (1-2+) relative to the background expression of the nonneoplastic cells inside or adjacent to the neoplastic lesions (0-1+) (Figure 1). Significantly higher expression was observed in patients 2, 3, 4, and 5 than in patients 1 and 6. No apparent difference was observed between the primary tumors and corresponding lymph nodule metastases.

### 3.3. Two Hotspot Mutations H1047R and E545K of the *PIK3CA* Gene Were Identified in Six SDC Patients

**Figure 2:**
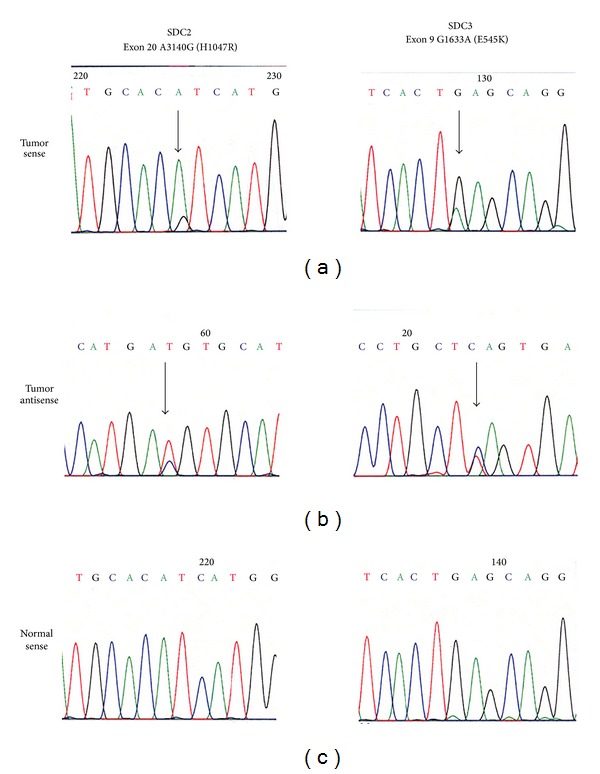
Two *PIK3CA* “hotspot” mutations were identified in the six SDC patients by genomic DNA sequencing. A hotspot mutation of the *PIK3CA* gene (exon 20 nucleotide 3140 A → G substitution) was identified in patient SDC 2. Another hotspot mutation of *PIK3CA* (exon 9 nucleotide 1633 G → A alteration) was detected in SDC3. Both mutations were confirmed by antisense sequencing and sequencing of an independent PCR product from the original genomic template, and not noted in the adjacent normal tissues.

To investigate whether genetic mutation is one of the molecular mechanisms that contributed to the elevated PIK3CA expression in SDC, we performed DNA sequencing analyses by microdissecting genomic DNAs from the neoplastic lesions and their adjacent normal tissues in these six SDC paraffin embedded specimens. Because it has been demonstrated that ~80% of the somatic mutations documented for *PIK3CA* clustered in the helical domain (exon 9) and kinase domain (exon 20) [12], here we only analyzed the DNA sequences of the exons 9 and 20 of the *PIK3CA* gene. The results showed that two hotspot mutations E545K and H1047R of the *PIK3CA* gene were identified in these six SDC specimens (33%). Hotspot mutation H1047R with a nucleotide 3140 A → G substitution in kinase domain at exon 20 of the* PIK3CA* gene was found in patient SDC2. Hotspot mutation E545K with a nucleotide 1633 G → A alteration in the helical domain at exon 9 of *PIK3CA* was identified in patient SDC3. Both mutations were confirmed by reverse sequencing and a second independent PCR sequencing (Figure 2). Moreover, these two mutations were only detected in the neoplastic lesions but not in their corresponding normal components (Figure 2), indicating that the identified mutations were somatic in nature. These two hotspot mutations have been previously shown to promote lipid kinase activity and enhance its downstream Akt signaling pathway [12, 21], suggesting their strong relevance in human tumorigenesis.

To investigate if the *PIK3CA* locus was amplified in those four SDC cases without *PIK3CA* mutations, we compared the copy numbers of the *PIK3CA* locus by quantitative PCR analysis of the neoplastic lesions and their corresponding normal tissues in each case. No more than 2-fold difference between the tumor and the corresponding normal components was detected in the four cases (see Supplementary Figure S1 available online at http://dx.doi.org/10.1155/2014/810487). This data suggest that the *PIK3CA* gene amplification was not a contributing molecular mechanism for the upregulation of the PIK3CA protein expression in SDC tumor cells.

**Figure 3:**
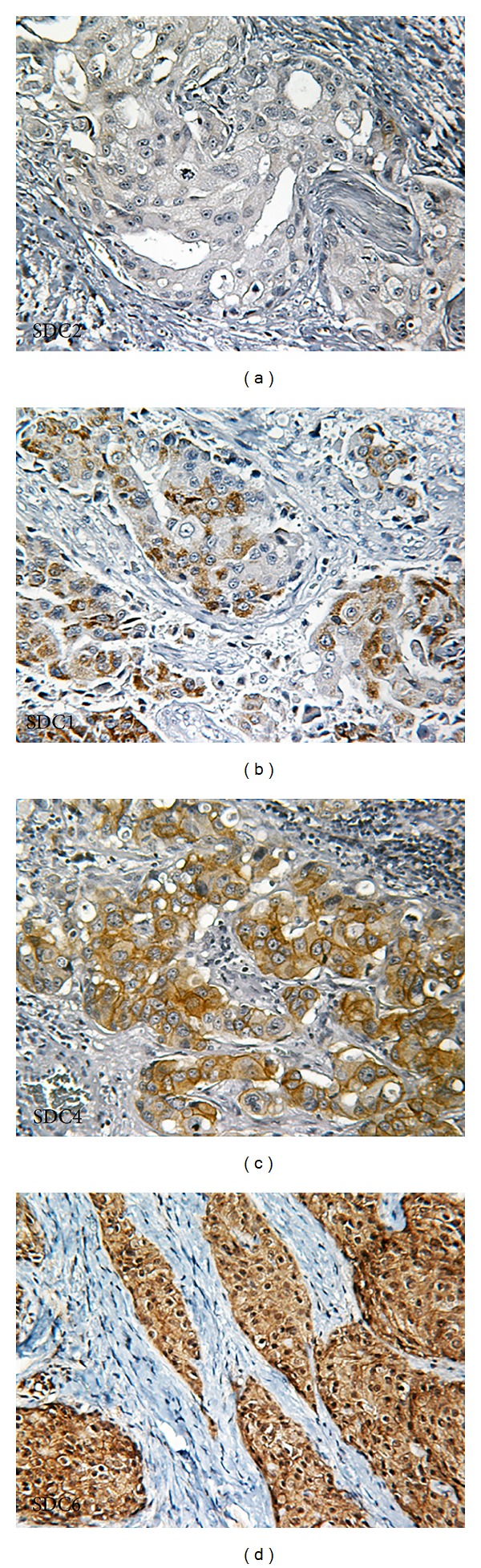
Overexpression of HER2 protein was detected in some of the salivary duct carcinoma samples. Three of the five patients, SDC1 (b), SDC4 (c), and SDC6 (d), displayed elevated HER2 expression, varying from + to +++. SDC2 was an example with HER2-negative staining (a). Magnification: 200x.

Frequent overexpression of HER2 protein has been reported in the human salivary ductal carcinoma [22]. To investigate if the *PIK3CA* mutations coexist with HER2 overexpression, we examined HER2 expression in the 5 of those 6 SDC cases by IHC. Three of five patients showed overexpression of HER2 from + to +++ (Figure 3). No coexistence of HER2 overexpression and *PIK3CA* mutation was observed among these five cases (Table 1).

## 4. Discussion

SDC is one of the most aggressive subtypes of salivary gland cancers [1]. Recently, elevated expressions of p53 and HER2/neu have been detected in some of SDC patients, which were shown to be associated with recurrence, poor prognosis, and distant metastasis [7, 23]. In this study, we found that oncogenic PIK3CA protein was significantly elevated in the salivary duct carcinoma of all six patients (Figure 1). Furthermore, we detected high incidence of *PIK3CA *hotspot mutations in SDC patients (33%) (4%, 78%; 95% confidence limits). The two *PIK3CA* hotspot mutations (E545 K and H1047R) are located at the helical domain and the kinase domain of the PIK3CA protein, respectively (Figure 2). These data suggest that oncogenic *PIK3CA* may play a critical role in the carcinogenesis of this rare disease, possibly from an early stage. These data are consistent with recent studies that reported *PIK3CA* mutations in patients with SDC [24, 25]. Moreover, Suzuki et al. have also reported positive staining of phosphorylated mTOR (a downstream gene of the PI3 K signaling) in 10/12 SDCs examined [26]. Combined with our previous studies that high incidence of *PIK3CA* gene mutations was identified in the other tumor types of human head and neck squamous cell carcinomas such as pharyngeal cancer [14, 15], the current study further confirmed the importance of oncogenic *PIK3CA* in the carcinogenesis of salivary duct epithelial cells and highlighted a critical oncogenic PI3 K signaling pathway in human head and neck cancers. It is conceivable that PI3 K pathway is frequently dysregulated in SDC and the activated *PIK3CA* function may have led to the reported increased p-mTOR expression.

Clinical data indicates that SDC2 with hotspot mutation H1047R in *PIK3CA* kinase domain was a 65-year-old male patient. SDC3 patient with hotspot mutation E545 K was the only female patient and was also diagnosed at 50 years-old, which is the youngest among this SDC cohort. Both patients with hotspot *PIK3CA* mutations were featured with perineural invasion and many lymph nodule metastases. Interestingly, SDC2 did not exhibit overexpression of HER2, while three of the four cases with wild-type *PIK3CA* displayed upregulated HER2 expression. Unfortunately no sufficient tissue remained from the SDC3 case for the HER2 IHC.

Oncogenic *PIK3CA* is mainly activated through gene amplification and “gain of function” single-nucleotide substitution in human cancers [27, 28]. In this study, all six SDC patients showed upregulated expressions of PIK3CA protein in tumor lesions, but only two of them harbored *PIK3CA* genetic mutations. Since gene amplification is a logical alternative molecular mechanism for *PIK3CA* activation [10, 27–29], we examined copy number alteration of *PIK3CA* by quantitative PCR. No amplification was detected in our samples; therefore, the source of activation is likely upstream of *PIK3CA*. Our data would recommend that when investigating activation of the PI3 K signaling pathway, other approaches such as IHC should be included to complement genomic DNA sequencing. Indeed, IHC for PIK3CA expression in cervical intraepithelial neoplasia has been shown possessing diagnostic significance for cervical cancer [30]. Thus, the PIK3CA and p-mTOR immunostaining results should be further examined for their high translational potentials as biomarkers for diagnosis/prognosis of human SDC or guiding target therapeutics for patients with SDC.

Despite aggressive surgical resection and postoperative adjuvant radiotherapy, the overall survival rate for SDC patients remains dismal. Clearly, more targets for chemotherapy or adjuvant therapy following surgery or in combination with radiotherapy are very desirable. Here we report high *PIK3CA* mutation rates in this rare disease with high mortality. Our data also suggests that nongenomic alteration is involved in the dysregulation of the PI3 K signaling pathway. In combination with other reports [24, 25], current data strongly support the notion that the PI3 K signaling pathway plays a critical oncogenic role in the development of human SDC and the prevalence of its dysregulation advocates its potential as a feasible therapeutic target.

## Supplementary Material

Fig. S1: No significant copy number change at the PIK3CA locus in the tumor lesions compared to the corresponding normal components was detected in the remaining four SDC cases without PIK3CA mutations. Relative copy numbers of the genomic PIK3CA locus were determined by quantitative real-time PCR and adjusted to the reference gene.

## Figures and Tables

**Table 1:** Clinical and pathologic features of salivary duct carcinoma cases.

Case	Gender	Age	Location	Tumor size	Perineural invasion	Margin	Lymph node	Pleomorphic adenoma	HER2 expression	PIK3CA mutation
SDC1	M	60	Parotid	2.3 cm	+	−	1/2	−	+	−
SDC2	M	65	Parotid	2.2 cm	+	+	15/29	−	−	H1047R
SDC3	F	50	Parotid	1.7 cm	+	−	8/44	+	N/A	E545K
SDC4	M	58	Parotid	2.0 cm	+	− (close)	14/45	+	++	−
SDC5	M	67	Parotid	N/A	N/A	+	0/12	+	−	−
SDC6	M	71	Parotid	1.6 cm	+	− (close)	N/A	−	+++	−

Abbreviations: N/A: not available or not applicable; close: less than 0.1 cm.

## Abbreviations

SDC: Salivary duct carcinoma
PCR: Polymerase chain reaction
PI3 K: Phosphatidylinositol 3-kinase
PIP3: Phosphatidylinositol-3, 4, 5-triphosphate.
