# Supplementary figures and images for: Oncogenic PIK3CA Mutation and Dysregulation in Human Salivary Duct Carcinoma
Source: Biomed Res Int. 2014 Jan 8;2014:810487. doi: 10.1155/2014/810487 (PMC3910486; doi:10.1155/2014/810487)

**Figure S1.**

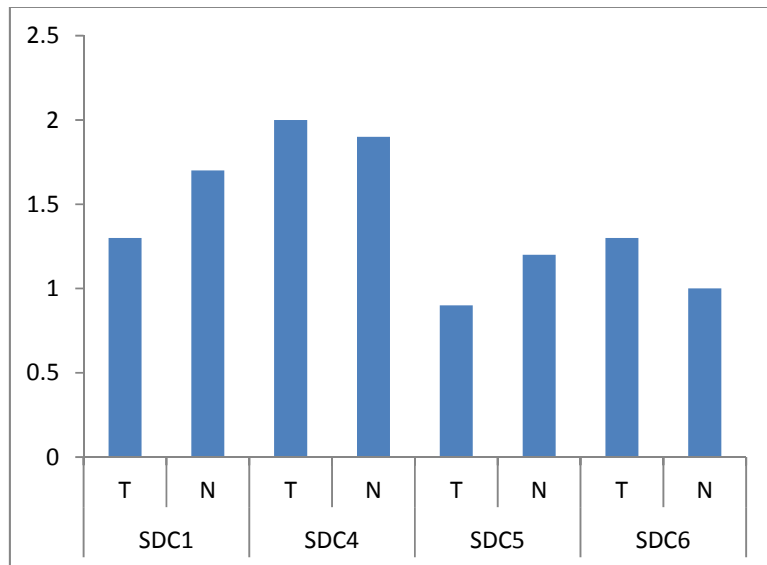

Supplement: Supplementary file 1 — Fig. S1: No significant copy number change at the PIK3CA locus in the tumor lesions compared to the corresponding normal components was detected in the remaining four SDC cases without PIK3CA mutations. Relative copy numbers of the genomic PIK3CA locus were determined by quantitative real-time PCR and adjusted to the reference gene. [file 810487.f1.pdf]
